# How and what adverse events are reported and captured in randomized control trials of emollients in the treatment of eczema?

**DOI:** 10.1093/ced/llad137

**Published:** 2023-04-19

**Authors:** Elizabeth R Emmett, Megan Allen, Sarah Crownshaw, Matthew J Ridd

**Affiliations:** Gloucestershire NHS Trust, Cheltenham, UK; Bristol Medical School, University of Bristol, Bristol, UK; Great Western Hospitals NHS Trust, Swindon, UK; Bristol Medical School, University of Bristol, Bristol, UK

## Abstract

**Background:**

Emollients are universally recommended for atopic dermatitis/eczema (‘eczema’), to improve the skin barrier and reduce symptoms. However, our knowledge of the frequency and nature of adverse effects associated with their use is limited.

**Objectives:**

We sought to determine how well adverse events are reported in randomized controlled trials (RCTs) of emollients for eczema.

**Methods:**

MEDLINE was searched from inception (1946) to May 2022. Inclusion criteria were RCTs of moisturizers or emollients used as a leave-on treatment (as the intervention or control) in adults or children with eczema. Exclusion criteria were non-RCTs; patients with other diagnoses included; use of emollient as bath additives, soap substitutes or as preventative; and not published in English. References of eligible papers were reviewed for any additional, relevant research. Data were extracted into an Excel spreadsheet and analysed descriptively. An assessment of study quality was carried out using the Joanna Briggs Institute tool for RCTs.

**Results:**

From 369 potential papers, 35 papers (reporting on 34 studies) were included. Most research was conducted in research centres or hospitals (unclear in 34%). In total, 89% reported collecting data on adverse events related to emollient treatment use but the methods used were poorly reported (40% unclear). Four papers used patient questionnaires/diaries. However, it was unclear how and what was collected as only two studies showed the questionnaires used.

**Conclusions:**

Reporting of adverse events related to emollient use in trials of patients with eczema is poor and inconsistent. Agreement should be reached on how and what adverse events should be collected, to standardize reporting across studies.

## Teaser

Emollients are universally accepted as a treatment for atopic dermatitis; however, our knowledge of the frequency and nature of adverse effects associated with their use is limited. We sought to determine how well adverse events are reported in randomized controlled trials of emollients for eczema. We concluded that they are poorly reported and agreement should be reached on how and what adverse events should be collected to standardize reporting across studies.

**What is already known about this topic?**

- Emollients use in people with eczema is determined by effectiveness and acceptability.
- Adverse events associated with emollient use are usually mild but estimates of their frequency vary widely.

**What does this study add?**

- Adverse event reporting in trials of emollients for eczema is poor and data are collected inconsistently across randomized controlled trials.
- Standardization of the methods of collecting and reporting emollient adverse effects would help with comparisons across different studies and products.

Atopic dermatitis/eczema (‘eczema’) is a chronic, inflammatory skin condition, affecting children and adults. Its global prevalence is steadily increasing.^1^ It is characterized by dry and itchy skin, prone to lichenification and skin infections.^2^ Emollients are recommended in the treatment of eczema; to soothe pruritis, to improve the skin’s barrier function and to help reduce the recurrence of disease flares.^1–4^ However, underuse is common and may be related to the acceptability of different products, including any adverse effects.^5^ A 2017 Cochrane review of randomized controlled trials (RCTs) comparing emollients in eczema reported that adverse events were only reported in half of studies (41/77), and these were uninformative.^1^ A more recent systematic review (2019) that included RCTs, cohort studies, case–control studies and case reports, also concluded that adverse events were poorly reported, with estimates of how common adverse events were widely varying (2–59% of patients).^6^

Extending the above work, the aim of this systematic review was to look at how RCTs captured data on adverse events associated with emollient use and what symptoms they discussed and reported on when they did.

## Materials and methods

The review was prospectively registered on Research Registry (unique number: reviewregistry1441) and conducted/reported in line with PRISMA guidance (Appendix S1; see the Supporting Information).^7^

### Literature search

The MEDLINE database (Ovid) was searched from 1946 to May 2022. The search strategy used is given in Appendix S2; see the Supporting Information.

The citations were exported into Microsoft Excel, and their eligibility assessed. Firstly, they were screened using the title and abstract. Next, the remaining papers were read in full to create a list of eligible papers. Lastly, these were then reviewed for any additional papers, which were then added to create the final list to be used for data extraction and analysis. Papers were independently screened by two authors (E.R.E. and S.C. or M.A.) and any questions were discussed with a fourth author (M.J.R.) until a decision was made.

### Quality assessment

An overall quality assessment of the papers included was carried out using the Joanna Briggs Institute checklist for RCTs.^8^ M.A. led this assessment and ambiguities were resolved with E.R.E. and M.J.R.

### Inclusion and exclusion criteria

Inclusion criteria were: RCTs of moisturizers or emollients used as a leave-on treatment (as the intervention or control) in adults or children with atopic eczema or atopic dermatitis. Papers were excluded if: they did not study leave-on emollients or moisturizers (e.g. bath additives or soap substitutes); were not RCTs; included patients with other diagnoses; the use of emollients as preventative treatments; were not published in English; and were not in humans.

Emollients or moisturizers used in the treatment of eczema do not have a strict definition, but for the purposes of this review were defined as products applied directly to the skin to help retain moisture. They did not contain recognized anti-inflammatory agents such as topical corticosteroids or calcineurin inhibitors.

The adverse events focused on in this report are those related to the treatment.

### Data extraction

Microsoft Excel was used to extract and analyse the data. One author, E.R.E, piloted the data extraction tables, before they were finalized. E.R.E. first completed the data extraction then, a second author, M.A., independently checked them. Discrepancies were discussed with a third author, M.J.R. The main outcomes assessed were: how many studies reported on adverse events, if and how those studies collected these data and what the adverse events as a result of emollients were. Data extraction focused on treatment-related adverse events, specifically localized skin reactions. Data about the study characteristics were also collected including: patient demographics, setting, type of emollient and if they reported broad or detailed data on adverse events.

We collected data on any of the treatment-related adverse events because of either the emollient used as the control or intervention on the assumption (where not stated) that the same methodology was used in all trial arms.

## Results

### Literature search

**Figure 1:**
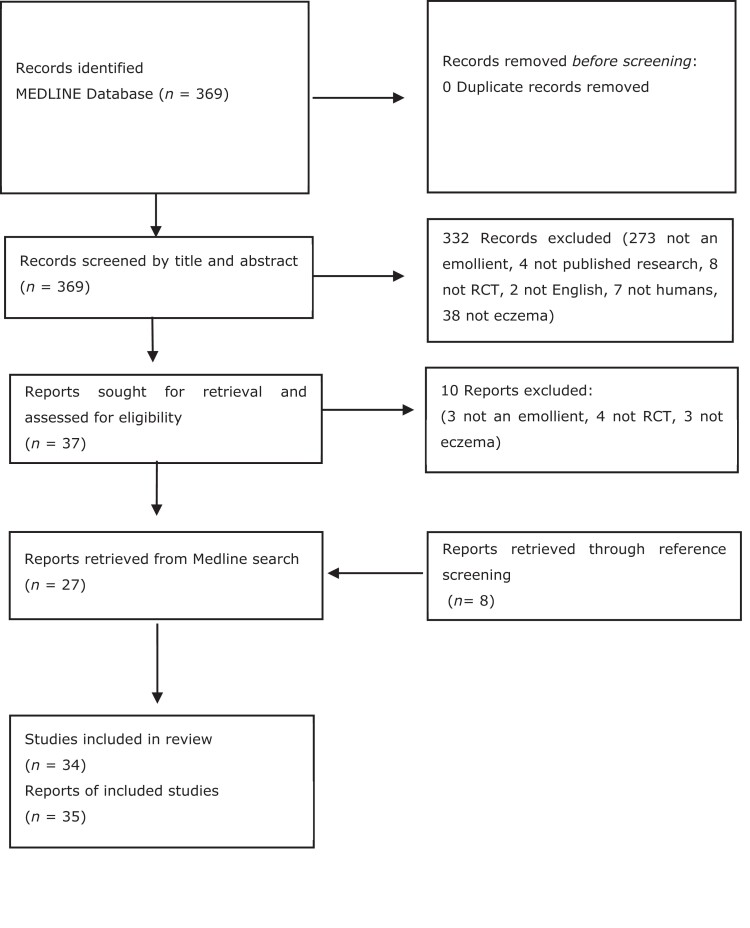
PRISMA flowchart – eligibility. RCT, randomized controlled trial.

From 369 results, 332 papers were excluded. In total, 37 papers were retrieved in full, and a further 10 papers excluded. Eight additional articles were included after screening the references of eligible papers, creating 35 as the final list of eligible reports, or 34 studies (see Figure 1).

### Study characteristics

The 35 papers were published from 1977 to 2021 (Table S1; see Supporting Information). The majority, 43% (15/35) were focused on paediatric patients^9–23^ compared with 26% (9/35) on adults^24–32^ and 31% (11/35) that included both.^33–43^ Four (11%) were conducted in multiple countries^12,13,24,41^ and 14% (5/35) did not clearly state where the research took place.^15,17,27,30,38^ Of those conducted in one country, 20% (7/35) were in the USA^11,18,22,23,34–36,43^ and 17% (6/35) in Germany.^10,16,26,29,32,33^

The research settings were primarily in research centres (37%) 13/35^11–13,18,19,26,29,30,32,35,36,39,41^ or hospitals (26%) 9/35^9,10,16,24,25,28,31,40,42^ and one took place across both (3%).^23^ There was no research in a community setting, although 12 papers (34%) were unclear.^14,15,17,20–22,27,33,34,37,38,43^

Five studies were within-patient studies, where both the emollient and control were used on the same patient.^15,27,29,35,43^

### Assessment of overall study quality

In 57% (20/35) of the papers studied, allocation was either not concealed or it was unclear.^9,14,16,18,20–22,24,26–28,30–34,37,39,41,43^ In 26% (9/35) of the total papers included, patients were not blind to allocation^9,10,12,13,15,16,29,33,35^ and for 9% (3/35) it was unclear if any participants were blind to allocation or not.^19,21,27^ In two papers – one using a patient diary^10^ and another a questionnaire^33^ – the participants were not blind to allocation. In 14% (5/35) the assessor was not blind to allocation^9,12,13,16,28^ and in 9% (3/35) it was unclear.^19,21,27^ In three papers with investigator assessed data collection, the investigators were not blinded.^12,16,28^

In all but one of the papers^31^ it was unclear whether outcomes were measured in a reliable way as the number and training of the assessors was not mentioned (Table S2; see Supporting Information).

### Emollients studied

The 35 papers included 46 different emollients (Appendix S3; see Supporting Information). Atopiclair^TM^ was the most commonly used emollient (three papers).^13,18,21^ The emollients used included a range of formulations, the most common was cream (41%; 19/46)^10–14,16,18,21,22,24,25,27,31,32,34,35,38,39,42,43^ followed by lotion (13%; 6/46).^9,15,23,26,30,43^ The ingredients in the emollients varied from botanicals, ceramides and ‘active’ ingredients (Appendix S3).

### Adverse events data capture and methodology

The majority of reports, 89% (31/35), did capture data on adverse events related to the treatment, although only 58% (18/31) provided a specific report into the nature of the adverse events experienced. The other papers provided a broad summary of adverse events explaining either there were no adverse events experienced or highlighting that the product was deemed safe.

The methods used to capture the data on adverse events were poorly explained. Most reports (14/35; 40%) were unclear.^9,13,15,17,21,22,25,29,30,32,34,38,42,43^ Where stated, 26% (9/35) were investigator assessed,^12,16,23,24,28,35,36,40,41^ 6% (2/35) were patient reported^14,18^ and 1 study^39^ used both the investigator and the patient to capture adverse event data (Table 1).

**Table 1:** Methodology and reporting on adverse events (AEs) (*n* = 35)

Methodology	Studies using this methodology, *n*	Reported on the nature of AEs experienced, *n* (%)
Investigator assessed	9	6 (67)
Investigator assessed and patient reported	1	1 (100)
Patient case report forms	1	1 (100)
Patient diaries	1	0 (0)
Patient reported	2	2 (100)
Questionnaire	3	1 (33)
Unclear	14	7 (50)
No data collection	4	0 (0)

A range of other methods were mentioned such as patient diaries,^10^ case report forms,^11^ and questionnaires.^19,20,33^ Two studies shared their questionnaire on adverse events.^19,20^ One focused on the frequency of adverse events,^20^ the other had a graph, which showed the question used for data capture (‘How bothered were you by the study regime’s side effects?’) and the results.^19^ Angelova-Fischer *et al.* did not share their questionnaire, but present the results of the frequency and nature of the adverse events for the three products used in the trial.^33^

### Nature of adverse events

In 31% (11/35) of the studies, one or more patients stopped using the product because of treatment-related adverse events.^13,14,16,18,21,23,24,28,30,32,41^ In each of these studies a more detailed report of adverse events experienced was reported. In five of the studies, it was unclear if patients stopped using the product or not.^17,20,26,29,31^

**Figure 2:**
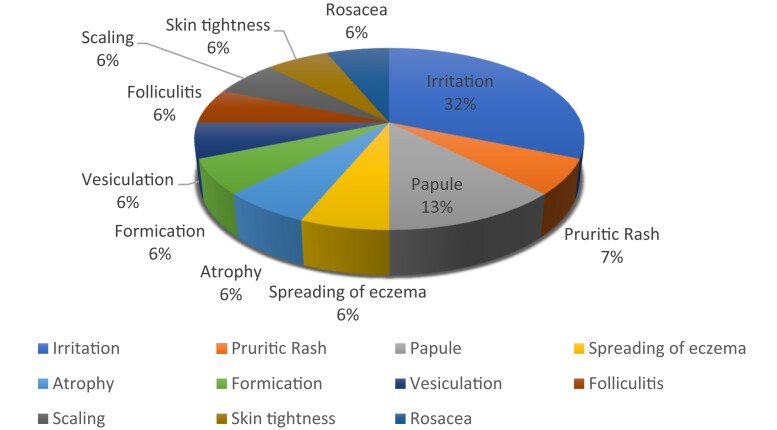
Breakdown of ‘other’ adverse symptoms.

Where studies did provide information on the type of side-effects experienced from the emollient the most common were ‘pruritis’,^11,12,14,16,24,25,30,32,33,41,42^ ‘burning on application’^12,14,18,21,23,24,32,33,41^ and ‘erythema’ (Table 2).^12,14,16,23,24,​28,30,32,33^ Eleven studies mentioned ‘other’ symptoms, of these ‘irritation’ was the most common, followed by ‘papule’ (Figure 2).^9,11,14,16,23,24,28,32,33,38,41^

**Table 2:** Symptoms reported as adverse events (*n* = 35)

Symptom	Papers reporting, *n* (%)
Itching/pruritis	11 (31)
Erythema	9 (26)
Burning or warm on application	9 (26)
Stinging on/after application	6 (17)
Worsening of eczema/flare ups	6 (17)
Allergic reactions (e.g. contact dermatitis/hypersensitivity)	2 (6)
Skin infections	2 (6)
Dryness	2 (6)
New rash	2 (6)
Pain	1 (3)
Swelling	1 (3)
Tingling	0 (0)
Peeling of skin	0 (0)

## Discussion

We identified 35 papers reporting on 34 trials of 46 emollients. They primarily included children and took place in research centres and secondary care sites. Four papers did not discuss adverse events at all,^26,27,31,37^ and of the remaining 31 papers, 58% provided detail on the adverse events experienced. The methodology to collect data on adverse events was unclear in 40% of the papers and only two papers shared their data-collection tools. Where studies provided detailed information about the type of adverse events experienced with emollients, pruritis, erythema and burning on application were the most common, whereas ‘irritation’ was the most commonly mentioned ‘other’ symptom.

This study has some limitations in addition to its strengths. To the best of our knowledge, this review is the first to focus on how and what adverse events are reported in trials of emollients used in the treatment of eczema. Because of the lack of consensus about how or what symptoms should be sought and reported, and variation across countries and cultures in how terms are understood or used, there is likely to be inconsistency in the use of terminology. For instance, one study may have referred to ‘stinging’ and another study may have referred to ‘burning’ in reference to the same side-effect. The generally poor reporting of adverse events was also reflected in poor reporting generally, as noted by the overall quality assessment.

This review collected data on the adverse events that were reported. If adverse events were experienced but there was no method to collect this information then it would have been missed. This review was also limited by only including papers published in English and only searching one database (MEDLINE). This review only focused on RCTs, which means that other study types with relevant information will have been missed.

The van Zuuren *et al.* Cochrane review of trials comparing moisturizers in the treatment of eczema, concluded that adverse event reporting for emollients needed to be more ‘complete’.^1^ This more in-depth assessment highlights a lack of consistency in the methodology used to collect data on adverse events.

Previous research has also highlighted that adverse events were generally mild in nature and uncommon with emollients and moisturizers.^1,6^ Yet 31% of the papers in this review had a participant stop using a product because of treatment-related side-effects. Adverse events maybe mild but they are not insignificant. It is therefore important that adverse events are accurately recorded and reported to improve clinical practice. Since running the searches, the Best Emollients for Eczema (BEE) trial has been published,^44^ which reported that 37% of children experienced one or more adverse event, although (with the exception of stinging) this did not differ between lotions, creams, gels or ointments.

A study looking at unwanted side-effects of emollients also found stinging or burning on application to be one of the more commonly experienced adverse events; however, this symptom could be considered a normal response to an emollient and associated with the severity of the eczema rather than an adverse event.^45^

## Conclusions

This review highlights a lack of consistency in the methods used to assess and report on adverse events. One way to reduce the variability in methodology would be to agree on a list of events to be routinely reported and create standardized tools to aid their collection. The BEE trial summarized the findings from patient-completed questionnaires by symptom and emollient type, clearly demonstrating the frequency and nature of the adverse event.^44^ This was similar to Angelova-Fischer *et al.* but included a more extensive list of symptoms such as worsening of eczema, peeling of skin and swelling.^33,44^

Further research into the nature of adverse events from use of emollients would be welcome to help shed light on what could be considered a ‘normal’ but unpleasant effect of an emollient on the skin and what is an adverse event. This will improve the ability of clinicians to educate patients as to what to expect from their emollient and hopefully improve adherence to treatment.

Adverse events in RCTs about emollients used in the treatment of eczema are poorly reported. The methodology used to collect data on adverse events varies and often was unclear. Improving the quality of data collection and reporting using standardized tools could help improve our understanding of the relative merits of different emollients.

## Supplementary Material

llad137_Supplementary_Data

## Data Availability

Data are available on request from the corresponding author.
