# Supplementary material for: How and what adverse events are reported and captured in randomized control trials of emollients in the treatment of eczema?
Source: Clin Exp Dermatol. 2023 Apr 19;48(8):889–94. doi: 10.1093/ced/llad137 (PMC10359396; doi:10.1093/ced/llad137)
Supplement: llad137_Supplementary_Data [file llad137_supplementary_data.docx]

# Appendix 1: Search terms used in Medline database

1 "adverse reaction*".mp. or exp "Drug-Related Side Effects and Adverse Reactions"/

2 "side effect*".mp.

3 "adverse effect*".mp.

4 "adverse event*".mp.

5 "side effect*".ab,ti.

6 exp Emollients/ or emollient*.mp.

7 moisturiser*.mp.

8 moisturizer*.mp.

9 exp Eczema/ or eczema.mp.

10 "atopic dermatitis".mp. or exp Dermatitis, Atopic/

11 "Emollients"/ae [Adverse Effects]

12 exp Skin Cream/ or exp Ointments/

13 lubricant*.mp.

14 ointment*.mp.

15 9 OR 10

16 1 or 2 or 3 or 4 or 5

17 6 or 7 or 8 or 11 or 12 or 13 or 14

18 15 and 16 and 17

19 limit 18 to (adaptive clinical trial or clinical study or clinical trial, all or clinical trial, phase i or clinical trial, phase ii or clinical trial, phase iii or clinical trial, phase iv or clinical trial or comparative study or controlled clinical trial or evaluation study or multicenter study or observational study or pragmatic clinical trial or randomized controlled trial or twin study or validation study)

# Appendix 2 - Study Characteristics

| Reference | Year of Publication | Country | Setting | Number of Participants Randomised | Adult or Paediatric | Patient Age Group (years) | Within patient study |
| --- | --- | --- | --- | --- | --- | --- | --- |
| [Åkerström U et al (2015)](https://www.medicaljournals.se/acta/content_files/files/pdf/95/5/4351.pdf) ^24^ | 2015 | Finland, Norway, Sweden | Hospital | 172 | Adult | 18 - 82 | NO |
| [Angelova-Fischer I et al (2014)](https://onlinelibrary.wiley.com/doi/10.1111/jdv.12479)^33^ | 2014 | Germany | Unclear | 20 | Both | 12 - 65 | NO |
| [Angelova-Fischer I et al (2018)](https://www.medicaljournals.se/acta/content/abstract/10.2340/00015555-2882) ^26^ | 2018 | Germany | Research Centre | 26 | Adult | 19 - 64 | NO |
| [Bissonnette R et al (2010)](https://onlinelibrary.wiley.com/doi/10.1111/j.1473-2165.2010.00476.x) ^30^ | 2010 | Unclear | Research Centre | 100 | Adult | 18 - 70 | NO |
| [Boguniewicz M et al (2008)](https://pubmed.ncbi.nlm.nih.gov/18492531/) ^12,18^ | 2008 | USA | Research Centre | 142 | Paediatric | 0.5- 12 | NO |
| [Boralevi F et al (2014)](https://onlinelibrary.wiley.com/doi/10.1111/jdv.12314) ^12^ | 2014 | France, Estonia, Lithuania, Poland , Romania | Research Centre | 251 | Paediatric | 2 - 6 | NO |
| Draelos ZD et al (2019) ^22^ | 2019 | USA | Unclear | 39 | Paediatric | 3 - 18 | NO |
| Draelos ZD et al (2019) ^34^ | 2019 | USA | Unclear | 108 | Both | 3 - 73 | NO |
| Emer JJ et al (2011) (^35^ | 2011 | USA | Research Centre | 20 | Both | 14 - 50 | YES |
| Frankel A et al 2011 (^36^ | 2011 | USA | Research Centre | 30 | Both | 4- 69 | NO |
| [Gayraud F et al (2015)](https://onlinelibrary.wiley.com/doi/10.1111/jocd.12145) ^14^ | 2015 | Poland | Unclear | 123 | Paediatric | 0.5 - 5 | NO |
| [Giordano-Labadie F et al (2009)](https://www.tandfonline.com/doi/pdf/10.1080/09546630600552216?needAccess=true) ^20^ | 2009 | France | Unclear | 76 | Paediatric | 6 - 12 | NO |
| [Haider SA (1977)](https://www.ncbi.nlm.nih.gov/pmc/articles/PMC1607321/pdf/brmedj00467-0020.pdf) ^17^ | 1977 | Unclear | Unclear | 42 | Paediatric | 0 - 14 | NO |
| [Khiljee S et al (2015)](http://www.pjps.pk/wp-content/uploads/pdfs/28/6/Paper-14.pdf) ^37^ | 2015 | Pakistan | Unclear | 360 | Both | Unclear | NO |
| [Korting HC et al (2010)](https://onlinelibrary.wiley.com/doi/10.1111/j.1468-3083.2010.03616.x) ^16^ | 2010 | Germany | Hospital | 99 | Paediatric | 0 - 12 | NO |
| [Kwon SH et al (2019)](https://www.tandfonline.com/doi/full/10.1080/09546634.2018.1544407) ^38^ | 2019 | Unclear | Unclear | 43 | Both | 4 - 40 | NO |
| [Lee et al (2008)](https://onlinelibrary.wiley.com/doi/full/10.1111/j.1346-8138.2008.00565.x?saml_referrer) ^39^ | 2008 | Korea | Research Centre | 21 | Both | 5 - 28 | NO |
| [Lin YK et al (2020)](https://www.sciencedirect.com/science/article/pii/S0378874119327898) ^40^ | 2020 | Taiwan | Hospital | 48 | Both | 6 - 65 | NO |
| [Lisante TA et al (2017)](https://www.tandfonline.com/doi/pdf/10.1080/09546634.2017.1303569?src=getftr) ^9,11^ | 2017 | USA | Research Centre | 90 | Paediatric | 0.5 - 18 | NO |
| [Liu L et al (2018)](https://www.tandfonline.com/doi/full/10.1080/09546634.2017.1401211) ^9,27^ | 2018 | China | Hospital | 107 | Paediatric | 1 - 17 | NO |
| [Ma L et al (2017)](https://pubmed.ncbi.nlm.nih.gov/29143926/) ^19^ | 2017 | China | Research Centre | 64 | Paediatric | 2 - 12 | NO |
| [Nistico SP et al (2017)](https://onlinelibrary.wiley.com/doi/full/10.1111/dth.12523?saml_referrer)^27^ | 2017 | Unclear | Unclear | 22 | Adult | 18 + | YES |
| [Patrizi A et al (2008)](https://onlinelibrary.wiley.com/doi/10.1111/j.1399-3038.2008.00724.x) ^21^ | 2008 | Italy | Unclear | 60 | Paediatric | 2 - 17 | NO |
| [Peserico A et al (2008)](https://onlinelibrary.wiley.com/doi/10.1111/j.1365-2133.2008.08436.x) ^41^ | 2008 | Germany, Italy, Spain | Research Centre | 221 | Both | 12 + | NO |
| [Simpson E et al (2012)](https://www.tandfonline.com/doi/full/10.3109/09546634.2012.713461) ^29^ | 2013 | Germany | Research Centre | 20 | Adult | 18 - 65 | YES |
| [Spada, Fabrizio et al (2021)](https://www.ncbi.nlm.nih.gov/pmc/articles/PMC8459234/) (^25,46^ | 2021 | Australia | Hospital | 100 | Adult | 18 - 73 | NO |
| [Stainer R et al (2005)](https://onlinelibrary-wiley-com.nottingham.idm.oclc.org/doi/pdf/10.1111/j.1365-2133.2004.06303.x) | 2005 | UK | Hospital and Research Centre | 114 | Paediatric | 2 - 12 | NO |
| [Stettler H et al (2017)](https://www.tandfonline.com/doi/pdf/10.1080/09546634.2017.1328938?src=getftr) ^10^ | 2017 | Germany | Hospital | 108 | Paediatric | 0 -4 | NO |
| [Stucker, M et al (2004)](https://onlinelibrary.wiley.com/doi/full/10.1111/j.1365-2133.2004.05866.x?sid=nlm%3Apubmed) ^32^ | 2004 | Germany | Research Centre | 49 | Adult | 18 - 70 | NO |
| Tamura M at al (2015) ^28^ | 2015 | Japan | Hospital | 50 | Adult | 20 - 65 | NO |
| [Tan WP (2010)](https://onlinelibrary.wiley.com/doi/full/10.1111/j.1365-2230.2009.03719.x?saml_referrer) ^13,42^ | 2010 | Singapore | Hospital | 60 | Both | 12 - 40 | NO |
| [Tiplica GS et al (2017)](https://onlinelibrary.wiley.com/doi/10.1111/pde.13113) ^13^ | 2017 | France, Estonia, Lithuania, Poland,Romania | Research Centre | 335 | Paediatric | 2 - 6 | NO |
| [Udompataikul M et al (2010)](https://onlinelibrary.wiley.com/doi/10.1111/j.1468-3083.2010.03845.x) ^15^ | 2010 | Unclear | Unclear | 30 | Paediatric | 2 - 15 | YES |
| [Wirén K et al (2009)](https://onlinelibrary.wiley.com/doi/full/10.1111/j.1468-3083.2009.03303.x?saml_referrer)  ^31^ | 2009 | Sweden | Hospital | 55 | Adult | 18 - 65 | NO |
| Zirwas M et al (2017) ^43^ | 2017 | USA | Unclear | 1. 34 & 2. 32 | Both | 11 + | YES |

# Appendix 3 – Quality of study assessment

| Name of paper | Randomised to groups? | Allocation concealed? | Groups similar at baseline? | Participants blind? | Delivering treatment blind? | Assessors blinded? | Identical treatment? | Follow up complete? | Analysed in split groups? | Outcomes measured in same way? | Outcomes measured in a reliable way? | Appropriate statistical analysis? | Trial design appropriate? |
| --- | --- | --- | --- | --- | --- | --- | --- | --- | --- | --- | --- | --- | --- |
| [Åkerström U et al (2015)](https://www.medicaljournals.se/acta/content_files/files/pdf/95/5/4351.pdf) | Y | U | U | Y | Y | Y | Y | Y | Y | Y | U | Y | Y |
| [Angelova-Fischer I et al (2014)](https://onlinelibrary.wiley.com/doi/10.1111/jdv.12479) | Y | U | Y | N | N | Y | Y | Y | Y | Y | U | Y | Y |
| [Angelova-Fischer I et al (2018)](https://www.medicaljournals.se/acta/content/abstract/10.2340/00015555-2882) | Y | U | Y | Y | Y | Y | Y | Y | Y | Y | U | Y | Y |
| [Bissonnette R et al (2010)](https://onlinelibrary.wiley.com/doi/10.1111/j.1473-2165.2010.00476.x) | Y | U | Y | Y | Y | Y | Y | Y | Y | Y | U | Y | Y |
| [Boguniewicz M et al  (2008)](https://pubmed.ncbi.nlm.nih.gov/18492531/) | Y | U | Y | Y | Y | Y | Y | Y | Y | Y | U | Y | Y |
| [Boralevi F et al (2014)](https://onlinelibrary.wiley.com/doi/10.1111/jdv.12314) | Y | Y | Y | N | N | N | Y | Y | Y | Y | U | Y | Y |
| Draelos ZD et al (2019) | Y | U | N | Y | Y | Y | Y | Y | Y | Y | U | Y | Y |
| Draelos ZD et al (2019) | Y | U | U | Y | Y | Y | Y | Y | Y | Y | U | Y | Y |
| Emer JJ et al (2011) | Y | Y | Y | N | N | Y | Y | Y | Y | Y | U | Y | Y |
| Frankel A et al 2011 | Y | Y | Y | Y | Y | Y | Y | Y | Y | Y | U | Y | Y |
| [Gayraud F et al (2015)](https://onlinelibrary.wiley.com/doi/10.1111/jocd.12145) | Y | U | Y | Y | Y | Y | Y | Y | Y | Y | U | Y | Y |
| [Giordano-Labadie F et al (2006)](https://www.tandfonline.com/doi/pdf/10.1080/09546630600552216?needAccess=true) | Y | U | Y | Y | Y | Y | Y | Y | Y | Y | U | Y | Y |
| [Haider SA (1977)](https://www.ncbi.nlm.nih.gov/pmc/articles/PMC1607321/pdf/brmedj00467-0020.pdf) | Y | Y | Y | Y | Y | Y | Y | Y | Y | Y | U | Y | Y |
| Khiljee S et al (2015) | Y | U | U | Y | y | Y | Y | Y | Y | Y | U | N | Y |
| [Korting HC et al (2010)](https://onlinelibrary.wiley.com/doi/10.1111/j.1468-3083.2010.03616.x) | Y | N | Y | N | N | N | Y | Y | Y | Y | U | Y | Y |
| [Kwon SH et al (2019)](https://www.tandfonline.com/doi/full/10.1080/09546634.2018.1544407) | Y | Y | N | Y | Y | Y | Y | Y | Y | Y | U | Y | Y |
| [Lee et al (2008)](https://onlinelibrary.wiley.com/doi/full/10.1111/j.1346-8138.2008.00565.x?saml_referrer) | Y | U | U | Y | Y | Y | Y | Y | Y | Y | U | N | Y |
| [Lin YK et al (2020)](https://www.sciencedirect.com/science/article/pii/S0378874119327898) | Y | Y | Y | Y | Y | Y | Y | Y | Y | Y | U | Y | Y |
| [Lisante TA et al (2017)](https://www.tandfonline.com/doi/pdf/10.1080/09546634.2017.1303569?src=getftr) | Y | Y | Y | Y | Y | Y | Y | Y | Y | Y | U | Y | Y |
| [Liu L et al (2018)](https://www.tandfonline.com/doi/full/10.1080/09546634.2017.1401211) | y | U | Y | N | N | N | Y | Y | Y | Y | U | Y | Y |
| [Ma L et al (2017)](https://pubmed.ncbi.nlm.nih.gov/29143926/) | Y | Y | U | U | U | U | Y | Y | Y | Y | U | Y | Y |
| [Nistico SP et al (2017)](https://onlinelibrary.wiley.com/doi/full/10.1111/dth.12523?saml_referrer) | Y | U | Y | U | U | U | Y | Y | Y | Y | U | Y | Y |
| [Patrizi A et al (2008)](https://onlinelibrary.wiley.com/doi/10.1111/j.1399-3038.2008.00724.x) | Y | U | Y | U | U | U | Y | Y | Y | Y | U | Y | Y |
| [Peserico A et al (2008)](https://onlinelibrary.wiley.com/doi/10.1111/j.1365-2133.2008.08436.x) | Y | U | Y | Y | Y | Y | Y | Y | Y | Y | U | Y | Y |
| [Simpson E et al (2012)](https://www.tandfonline.com/doi/full/10.3109/09546634.2012.713461) | Y | Y | Y | N | N | Y | Y | Y | Y | Y | U | Y | Y |
| [Spada, Fabrizio et al (2021)](https://www.ncbi.nlm.nih.gov/pmc/articles/PMC8459234/) | Y | Y | Y | Y | Y | Y | Y | Y | Y | Y | U | Y | Y |
| [Stainer R et al (2005)](https://onlinelibrary-wiley-com.nottingham.idm.oclc.org/doi/pdf/10.1111/j.1365-2133.2004.06303.x) | Y | Y | Y | Y | Y | Y | Y | Y | Y | Y | U | Y | Y |
| [Stettler H et al (2017)](https://www.tandfonline.com/doi/pdf/10.1080/09546634.2017.1328938?src=getftr) | Y | Y | Y | N | N | Y | Y | Y | Y | Y | U | Y | Y |
| [Stucker, M et al (2004)](https://onlinelibrary.wiley.com/doi/full/10.1111/j.1365-2133.2004.05866.x?sid=nlm%3Apubmed) | Y | U | U | Y | Y | Y | Y | Y | Y | Y | U | Y | Y |
| Tamura M at al (2015) | Y | U | Y | Y | Y | N | Y | Y | Y | Y | U | Y | Y |
| [Tan WP (2010)](https://onlinelibrary.wiley.com/doi/full/10.1111/j.1365-2230.2009.03719.x?saml_referrer) | y | Y | Y | Y | Y | Y | Y | Y | Y | Y | U | Y | Y |
| [Tiplica GS et al (2017)](https://onlinelibrary.wiley.com/doi/10.1111/pde.13113) | Y | Y | Y | N | N | N | Y | Y | Y | Y | U | Y | Y |
| [Udompataikul M et al (2011)](https://onlinelibrary.wiley.com/doi/10.1111/j.1468-3083.2010.03845.x) | Y | Y | Y | N | N | Y | Y | Y | Y | Y | U | Y | Y |
| [Wirén K et al (2009)](https://onlinelibrary.wiley.com/doi/full/10.1111/j.1468-3083.2009.03303.x?saml_referrer) | Y | U | Y | Y | Y | Y | Y | Y | Y | Y | Y | Y | Y |
| Zirwas M et al (2017) | Y | U | U | Y | Y | Y | Y | Y | Y | Y | U | U | Y |

# Appendix 4 – Emollients Evaluated

| Reference | Type of Emollient - Brand or Formulation (ingredient where known) | Formulation |
| --- | --- | --- |
| [Åkerström U et al (2015)](https://www.medicaljournals.se/acta/content_files/files/pdf/95/5/4351.pdf) | Canoderm cream 5% urea | Cream |
| [Angelova-Fischer I et al (2014)](https://onlinelibrary.wiley.com/doi/10.1111/jdv.12479) | o/w emollient containing licochalcone A (Glycyrrhiza Inflata root extract), decanediol (decylene glycol), menthoxypropanediol and ω-6-fatty acids  w/o emollient containing lichochalcone A and w-6-fatty acids | Unclear        Unclear |
| [Angelova-Fischer I et al (2018)](https://www.medicaljournals.se/acta/content/abstract/10.2340/00015555-2882) | Eucerin Atopicontrol Body Lotion (licochalcone A, omega-6 fatty acids, ceramide 3 and glycerol) | Lotion |
| [Bissonnette R et al (2010)](https://onlinelibrary.wiley.com/doi/10.1111/j.1473-2165.2010.00476.x) | Iso-Urea Moisturiser (5% Urea)  Lotion (10% Urea) | Unclear    Lotion |
| [Boguniewicz M et al (2008)](https://pubmed.ncbi.nlm.nih.gov/18492531/) | Atopiclair | Cream |
| [Boralevi F et al (2014)](https://onlinelibrary.wiley.com/doi/10.1111/jdv.12314) | Dexeryl (Glycerol 15%, liquid and soft paraffin 10%,) | Cream |
| Draelos ZD et al (2019) | Kamedis Eczema Therapy Cream (Combination of 6 botanicals) | Cream |
| Draelos ZD et al (2019) | Kamedis Eczema Therapy Cream (Combination of 6 botanicals) | Cream |
| Emer JJ et al (2011) | Eletone | Cream |
| Frankel A et al 2011 | Hylatopic emollient Foam | Foam |
| [Gayraud F et al (2015)](https://onlinelibrary.wiley.com/doi/10.1111/jocd.12145) | Atodermâ Intensive cream ( Vitamin B3, Zinc, Sucroesters) | Cream |
| [Giordano-Labadie F et al (2009)](https://www.tandfonline.com/doi/pdf/10.1080/09546630600552216?needAccess=true) | Exomega milk | Milk |
| [Haider SA (1977)](https://www.ncbi.nlm.nih.gov/pmc/articles/PMC1607321/pdf/brmedj00467-0020.pdf) | 10% sodium cromogylcate with white soft paraffin base ointment | Ointment |
| [Khiljee S et al (2015)](http://www.pjps.pk/wp-content/uploads/pdfs/28/6/Paper-14.pdf) | Micro emulsion, gel and ointment (5% Indian penny wort OR  walnut OR turmeric plant extract) | Emulsion, gel or ointment |
| [Korting HC et al (2010)](https://onlinelibrary.wiley.com/doi/10.1111/j.1468-3083.2010.03616.x) | Ichthosin (o/w cream with 4% sodiumbituminosulphonate) | Cream |
| [Kwon SH et al (2019)](https://www.tandfonline.com/doi/full/10.1080/09546634.2018.1544407) | PTPD-12 Moisturiser  Zeroid Intensive Ointment Cream | Cream    Cream |
| [Lee et al (2008)](https://onlinelibrary.wiley.com/doi/full/10.1111/j.1346-8138.2008.00565.x?saml_referrer) | Rosmarinic acid 0.3% with o/w cream (glycerin, methylparahydroxybenzoate, phenoxyethanol, xanthan gum, squalane, polysorbate 80, and beeswax) | Cream |
| [Lin YK et al (2020)](https://www.sciencedirect.com/science/article/pii/S0378874119327898) | Lindoil Emollient | Ointment |
| [Lisante TA et al (2017)](https://www.tandfonline.com/doi/pdf/10.1080/09546634.2017.1303569?src=getftr) | OTC 1% Oatmeal Cream  EpiCeram | Cream    Emulsion |
| [Liu L et al (2018)](https://www.tandfonline.com/doi/full/10.1080/09546634.2017.1401211) | Physiogel® lotion (o/w emulsion containing oils, shea butter, glycerin, squalane, hydrogenated lecithin and ceramide) | Lotion |
| [Ma L et al (2017)](https://pubmed.ncbi.nlm.nih.gov/29143926/) | Cetaphil Restoraderm Body Moisturizer | Unclear |
| [Nistico SP et al (2017)](https://onlinelibrary.wiley.com/doi/full/10.1111/dth.12523?saml_referrer) | Mavena® B12 barrier cream (0.07% Cyanocobalamin) | Cream |
| [Patrizi A et al (2008)](https://onlinelibrary.wiley.com/doi/10.1111/j.1399-3038.2008.00724.x) | Atopiclair  Atopiclair light | Cream    Cream |
| [Peserico A et al (2008)](https://onlinelibrary.wiley.com/doi/10.1111/j.1365-2133.2008.08436.x) | Advabase | Cream |
| [Simpson E et al (2012)](https://www.tandfonline.com/doi/full/10.3109/09546634.2012.713461) | Cetaphil Restoraderm Body Moisturizer | Unclear |
| [Spada, Fabrizio et al (2021)](https://www.ncbi.nlm.nih.gov/pmc/articles/PMC8459234/) | QV intensive with ceramides light moisturizing cream (ceramide) | Cream |
| [Stainer R et al (2005)](https://onlinelibrary-wiley-com.nottingham.idm.oclc.org/doi/pdf/10.1111/j.1365-2133.2004.06303.x) | Altoderm (5% sodium Cromoglicate) | Lotion |
| [Stettler H et al (2017)](https://www.tandfonline.com/doi/pdf/10.1080/09546634.2017.1328938?src=getftr) | NTP-CE, Bepanthen SensiDaily  Stelatopia Emollient Cream ( 2% sunflower oleodistillate) | Cream |
| [Stucker, M et al (2004)](https://onlinelibrary.wiley.com/doi/full/10.1111/j.1365-2133.2004.05866.x?sid=nlm%3Apubmed) | Regividerm (Vitamin B12 Cyanocobalamin 0.07%) | Cream |
| Tamura M at al (2015) | o/w type emulsion using a phospholipid emulsifier  o/w-type preparation containing 3.0 mg/g of a heparinoid substance | Emulsion |
| [Tan WP (2010)](https://onlinelibrary.wiley.com/doi/full/10.1111/j.1365-2230.2009.03719.x?saml_referrer) | Emollient (Triclosan) | Cream |
| [Tiplica GS et al (2017)](https://onlinelibrary.wiley.com/doi/10.1111/pde.13113) | Dexeryl (glycerol15% and liquid and soft paraffin 10%)  Atopiclair | Cream |
| [Udompataikul M et al (2010)](https://onlinelibrary.wiley.com/doi/10.1111/j.1468-3083.2010.03845.x) | Eucerin Soothing lotion (12% omega) | Lotion |
| [Wirén K et al (2009)](https://onlinelibrary.wiley.com/doi/full/10.1111/j.1468-3083.2009.03303.x?saml_referrer) | Canoderm cream 5% urea (o/w emulsion containing fractionated coconut oil, emulsifying wax, hydrogenated canola oil) | Cream |
| Zirwas M et al (2017) | CereVe Itch Relief Lotion (1% Pramoxine hydrochloride)  CereVe Itch Relief Cream (1% Pramoxine hydrochloride) | Lotion    Cream |
